# Changes in the Periodontal Gap After Long-Term Tooth Movement into Augmented Critical-Sized Defects in the Jaws of Beagle Dogs

**DOI:** 10.3390/dj12120386

**Published:** 2024-11-26

**Authors:** Kathrin Duske, Mareike Warkentin, Anja Salbach, Jan-Hendrik Lenz, Franka Stahl

**Affiliations:** 1 Department of Orthodontics, Rostock University Medical Centre, Strempelstrasse 13, 18057 Rostock, Germany; anja.salbach@kfo-salbach.de (A.S.); franka.stahl@med.uni-rostock.de (F.S.); 2 Working Group for Implant Materials, Faculty of Mechanical Engineering and Marine Technology, University of Rostock, Friedrich-Barnewitz-Strasse 4, 18119 Rostock, Germany; mareike.warkentin@uni-rostock.de; 3 Department of Oral and Maxillofacial Surgery, Rostock University Medical Centre, Strempelstrasse 13, 18057 Rostock, Germany; jan-hendrik.lenz@med.uni-rostock.de

**Keywords:** periodontal ligament, grafting materials, orthodontic tooth movement, bone regeneration

## Abstract

**Background/Objectives:** Extensive and closely coordinated remodeling processes take place in the periodontal ligament (PDL) and the adjacent bone during orthodontic tooth movement. In complex orthodontic cases, it is necessary to move teeth into an augmented bony defect, for example, in patients with cleft lip, alveolus, and palate. The important role of the PDL during tooth movement is well accepted but not fully understood. Therefore, the present study investigated the PDL after 23 weeks of tooth movement into an augmented critical-sized defect. **Methods:** The second molars of four beagle dogs were moved into a critical-sized defect, which was filled with bovine xenograft or nanocrystalline hydroxyapatite. Autogenous bone served as control. After 23 weeks, histological samples were microscopically analyzed, and the dimension of the PDL was measured. For statistical calculations, a Wilcoxon–Mann–Whitney test was used. **Results:** The PDL was significantly wider on the tension side compared with the compression side for all replacement materials analyzed (*p* ≤ 0.05). These results apply to both the mesial and distal roots. **Conclusions:** The remodeling processes reached equilibrium within 23 weeks, resulting in a wider gap on the tension side, which contrasts with the situation a few days after the initial force application.

## 1. Introduction

The movement of a tooth during orthodontic treatment is made possible by a series of tightly coordinated cellular processes, as well as the involvement of various mediators, through the application of a well-defined mechanical force [1]. Osteoblasts, osteoclasts, and osteocytes are involved in bone remodeling processes, which, according to the Biphasic Theory, occur on both the compression and tension side of the roots of an orthodontically moved tooth, with the Catabolic Phase preceding the Anabolic Phase [2]. During orthodontic tooth movement (OTM), the periodontal ligament (PDL) is compressed on the compression side [3,4] and stretched on the opposite side of the tooth root [4,5]. Currently, all of the mechanisms involved in this process are not fully understood [6], but the important role of the PDL in bone remodeling processes associated with OTM has been demonstrated [7].

During force application, an aseptic inflammation occurs [2,5], causing remodeling of the PDL [8] and bone [2]. The optimal distance between the tooth surface and the bone, provided by the PDL, is destroyed by the displacement of the tooth. The forces acting on the tooth can only be effectively distributed by an optimally organized PDL [9] that ensures optimum spacing. During OTM, the PDL undergoes significant dimensional changes, which differ between the compression and tension sides of the root. Several studies in rats and mice have investigated changes in PDL associated with OTM [5,10,11,12,13,14,15]. Some of these studies have compared the tension and compression sides and found a narrowing of the PDL on the compression side and a widening on the tension side [10,13,15]. Widening of the PDL was also found in studies comparing the compression side with the untreated control [5,12,14]. Obviously, the PDL is subject to pronounced changes in the first 1 to 14 days after the application of force. In rats, an increase in the PDL area on the tension side 24 h after the application of force was reported by Tsuge et al. [5]. The area decreased and almost returned to the untreated control level within the next 7 days. Laura et al. reported a similar trend within 14 days in rats and diabetes-induced rats. These were treated with insulin or insulin and metformin and showed a similar pattern of PDL values [13]. Rizk et al. examined the OTM and the bone remodeling processes in the alveolar bone of mice during a period of 5 weeks using a high-resolution micro-CT [9]. The study is one of the few to look at the PDL over a period of more than 3 weeks. The authors stated that it is difficult to distinguish between the compression and tension sides of a tooth in small animals during OTM. The PDL showed significantly greater thickness around the moved tooth compared with the untreated control [9].

Tooth movement into an augmented area is still under investigation, and the available results are inconsistent. Sun et al. summarized that bony reconstructions using material from the iliac crest stimulate bone remodeling [16]. Ru et al. compared bovine xenograft with a synthetic mixture of beta-tricalcium phosphate and hydroxyapatite. They concluded that the synthetic mixture leads to slower tooth movement, which is connected with less root resorption [17]. A comparison of autologous bone, human xenograft, and a synthetic bone substitute consisting of beta-tricalcium phosphate and hydroxyapatite resulted in similar distances of OTM over 57 days in rats [18].

The present study histologically investigated the changes in the PDL of teeth moved into critical-sized defects over a period of 23 weeks. This study was carried out in beagle dogs. Long-term tooth movement corresponds to clinical conditions. The data show that, within the first few days and weeks, fundamental changes in the PDL occur. The aim of this study was to investigate the dimensional changes in the PDL on the compression and tension sides of the tooth root, which was moved orthodontically.

## 2. Materials and Methods

**Figure 1:**
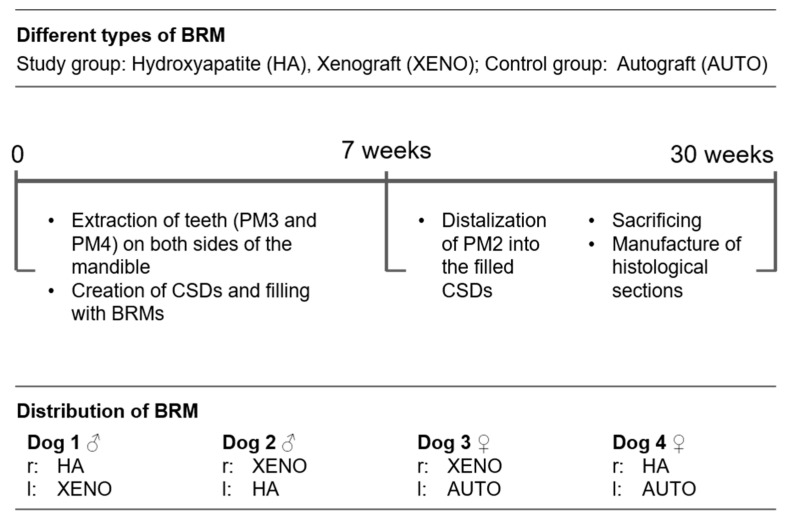
Schematic overview of the experimental design with different types of bone replacement materials (BRMs) used to fill critical-sized defects in the mandibles of four beagle dogs (CSD: critical-sized defect; PM: premolar; r: right side of the mandible; l: left side of the mandible; HA: hydroxyapatite; XENO: xenograft; AUTO: autograft).

A prospective animal study using beagle dogs was designed to address the study objective. Critical-sized defects in the mandible of beagle dogs, defined as bony defects that do not heal spontaneously [19], were filled with autogenous bone or two different bone replacement materials (BRMs). The second premolar (PM2), adjacent to the defect, was orthodontically moved into the filled area. After a long-term period of 23 weeks, the dimensional changes in the periodontal gaps of PM2 were investigated. The timeline of Figure 1 shows the steps of the present study design.

### 2.1. Surgical Procedure and Orthodontic Appliance

Animals: BRM were inserted in the mandibles of four beagle dogs (2 ♀, 2 ♂; BASF, Ludwigshafen, Germany). The experiments were conducted in accordance with the ARRIVE guidelines on animal research and were carried out during the year 2012. The Supplementary Materials include the ethics details (Ethics note). After surgical intervention, dogs were fed a soft diet. They had free access to fresh water. During the surgical sessions, dogs were anesthetized. For this purpose, each dog was sedated with acepromazine (10 mg/mL, 0.2 mL/10 kg body weight) and L-polamivet (1.0 mL/10 kg body weight). The administration of propofol (10 mg/mL, 3 to 4 mg/kg body weight) induced anesthesia. Isoflurane (1.1 to 1.8 Vol%) was added to the oxygen to achieve the anesthesia. The dogs were slowly awakened by discontinuing the inflow of isoflurane.

**Figure 2:**
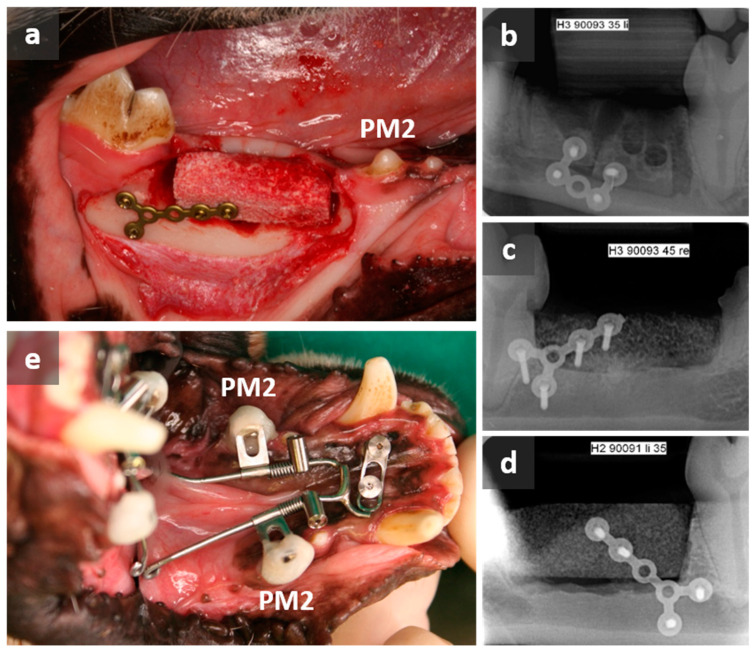
Intraoral images and X-rays of postoperative situations. Fixed BRMs positioned in the distal direction of the second premolar (PM2) (**a**). X-rays directly after surgical procedure of the autograft (**b**), xenograft (**c**), and hydroxyapatite (**d**). Bilateral distalization of PM2 started seven weeks after insertion of BRMs with an orthodontic appliance, which corresponded to the Beneslider system (**e**).

Experimental design: Critical-sized defects (dimensions in Table 1) were placed on the upper edge of the processus alveolaris mandibulae directly after the extraction of two premolars (PM3 and PM4). Besides the control group (AUTO), in which the removed autogenous bone was replaced, two study groups were formed, receiving either the nanocrystalline hydroxyapatite NanoBone^®^ (Artoss GmbH, Rostock, Germany) (HA) or the deproteinized bovine bone mineral BioOss^®^ (Geistlich Pharma, Wolhusen, Switzerland (XENO) to fill the critical-sized defects. The materials were randomly distributed (supported by the Institute for Biostatistics and Informatics in Medicine and Ageing Research; University Medical Centre, Rostock, Germany). All materials were fixed by using MPOS (2.0 Miniplate-osteosynthesis; Mondeal Company, Mühlheim, Germany) (Figure 2a). Additionally, two mini-screws were inserted into the anterior area of the mandible for later OTM. Finally, dental X-rays were taken (HF3000, Gierth X-Ray international GmbH, Riesa, Germany) (Figure 2b–d).

The second surgery occurred seven weeks after the placement of critical-sized defects and was performed using the same anesthesia protocol as described above. Bilateral distalization of PM2 into the augmented area was then initiated with an orthodontic appliance using skeletal anchorage (Figure 2e). The customized appliances corresponded to the Beneslider system (Benefit-System, PSM Medical Solutions, Tuttlingen, Germany) and were activated with 240 g (maximum compression). The system was not re-adjusted in the further course of the project. After a period of 23 weeks, the dogs were sacrificed, and mandibles were resected for histomorphometric analyses. They were then stored in artificial saliva (according to DAC/RF 7.5) supplemented with glutardialdehyde (2.5% final concentration) until further preparation.

### 2.2. Histomorphometric Preparation, Data Collection, and Analysis

The resected mandibles (*n* = 3) were embedded in cold-curing (4 °C) epoxy resin (EpoThin resin and hardener, Buehler, Germany). Afterward, they were sliced cross-sectionally to sections of 1 mm thickness (HistoSaw^®^ DDM-P 216; Medim, Giessen, Germany) under permanent water cooling. Then, the slice was ground into thin sections with a thickness of 20 µm (Tegra-Pol-15, Stuers, Willich, Germany) by using silicon carbide wet grinding paper (Buehler, Düsseldorf, Germany) with grain sizes of P-500 up to P-4000. After unstained microscopical analysis, thin sections were stained with toluidine blue and Giemsa solution according to the following protocol: 30 min staining with toluidine blue (Sigma Aldrich Chemie GmbH, Darmstadt, Germany), rinsing step with aqua dest., drying step with 100% ethanol, 20 min staining with azure eosin solution (Merck, Darmstadt, Germany), rinsing step with aqua dest., drying step with 100% ethanol. Thin polished and stained sections were investigated by light microscopy (SZX10, Olympus Germany, Hamburg, Germany) (LM) and scanning acoustic microscopy (SAM 300, PVA TePla GmbH, Wettenberg, Germany) (SAM). Ultrasound C-scans were taken from the top of each slice with a single-element 100 MHz ultrasound transducer. For this, cross sections were placed in a water bath.

#### 2.2.1. Periodontal Gap Measurements

**Figure 3:**
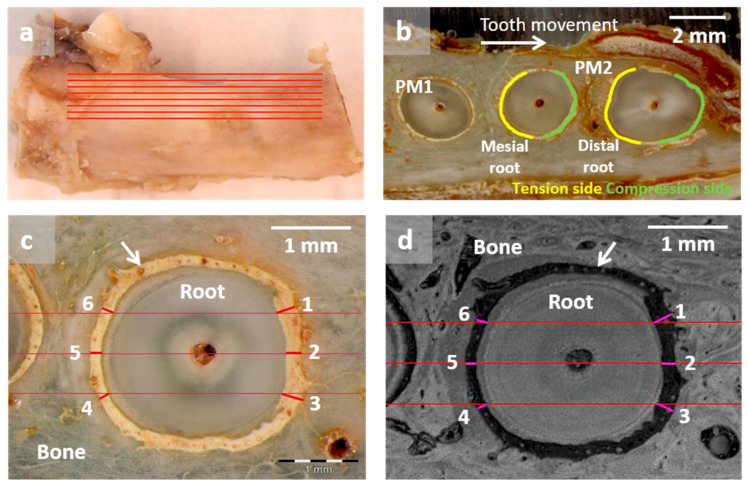
Resected mandible with marked slicing levels (**a**). Image (**b**) shows a slice with the root of the first premolar (PM1) and the mesial and distal root of the second premolar (PM2), which was moved orthodontically into the filled critical-sized defect. Starting from the direction of orthodontic tooth movement, the yellow lines in both roots of PM2 mark the tension side, and the green lines mark the compression side. Light microscopic (**c**) and scanning acoustic microscopic (**d**) images through the distal root of PM2, which were moved into the augmented critical-sized defect (Dog 3 left side). Arrows mark periodontal gaps, which were measured at three different points on the compression side (points 1–3) and at three different points on the tension side (points 4–6).

Unstained slices (Figure 3a) were used to measure the periodontal gaps of the mesial and distal root of PM2. In relation to the direction of the OTM, it was differentiated into the compression side (in the direction of the OTM) and the tension side (opposite direction of the OTM) (Figure 3b). The periodontal gap is the distance between root surface (cementum) and alveolar bone (arrows Figure 3c,d). On both sides, PDL widths were measured at three different points (numbers 1 to 6 in Figure 3c,d) (Gimp 2.10.6) with a vertical distance (horizontal red lines in Figure 3c,d) of 500 µm. The middle red line was adjusted to the same horizontal plane as the center of the root canal. With both microscopes (LM, SAM), the color distinctions between bone and PDL, as well as between PDL and tooth, were clearly visible. These analyses were performed with LM images (Figure 3c) and SAM images (Figure 3d).

#### 2.2.2. Proportion of Bone, Osteoid, and Bone Marrow

**Figure 4:**
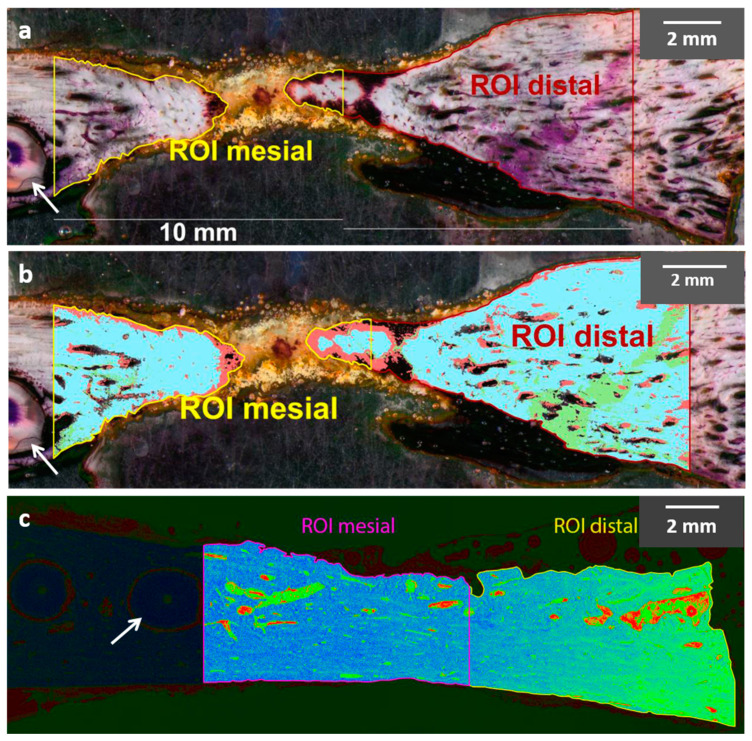
Light microscopic image of a stained thin section (Toluidine–Giemsa staining) with marked regions of interest (ROIs) (**a**). After histomorphometric analysis, bone is marked in light blue, cartilage matrix and osteoid in green, and bone marrow in red (**b**). Scanning acoustic microscopic image (**c**) of a thin section after histomorphometric analysis with marked ROIs (**b**). Arrows mark the roots of the PM2, which were moved into the augmented critical-sized defects.

Stained thin slices were used to evaluate the proportion of bone, osteoid, and bone marrow. For the measurement, images had to meet the following criteria: (a) sectional plane above the mandibular canal; (b) root of the PM2 was cut; (c) distal to the root was alveolar bone. Three to five images of each slice were arranged manually to obtain a panoramic image. This image included the PM2 and a distance of at least 20 mm in the distal direction (Figure 4). Two polygonal regions of interest (ROIs) were inserted and defined the mesial and distal ROIs. The first started directly behind the root of the PM2 at a distance of 10 mm. The distal ROI joined without a gap for another 10 mm (Figure 4). Both ROIs included the former critical size defect. The different reflection signal allows differentiation of the tissue in mineralized hard tissue (blue/phase A), unmineralized osteoid/cartilage matrix (green/phase B), and bone marrow (red/phase C).

For LM analysis, the commercial software Olympus cellSens Dimension V1.8.1 (Olympus Germany, Hamburg, Germany) was used to make an overall image out of several single pictures. These investigations were performed with LM and SAM.

### 2.3. Statistical Analysis

Data were analyzed with GraphPad Prism Version 6.01 (GraphPad Software, Inc., La Jolla, CA, USA). Descriptive statistics, including means and standard deviation, were calculated. The normality of the distribution was evaluated using the Shapiro–Wilk test. As variables appeared to be non-parametric, the Wilcoxon–Mann–Whitney test was used to find differences between the groups. Differences were considered statistically significant at *p* ≤ 0.05.

## 3. Results

The surgical procedure went without complications, and all dogs were able to feed on a soft diet, which was offered from the date of surgery. The healing process proceeded without complications. No specific events owing to critical-sized defects could be observed. Unfortunately, 21 days after the surgery, Dog 1 had his mandible fractured overnight, and physiological feeding was not possible anymore. This dog was euthanized immediately.

### 3.1. Macroscopic and Radiographic Results

**Figure 5:**
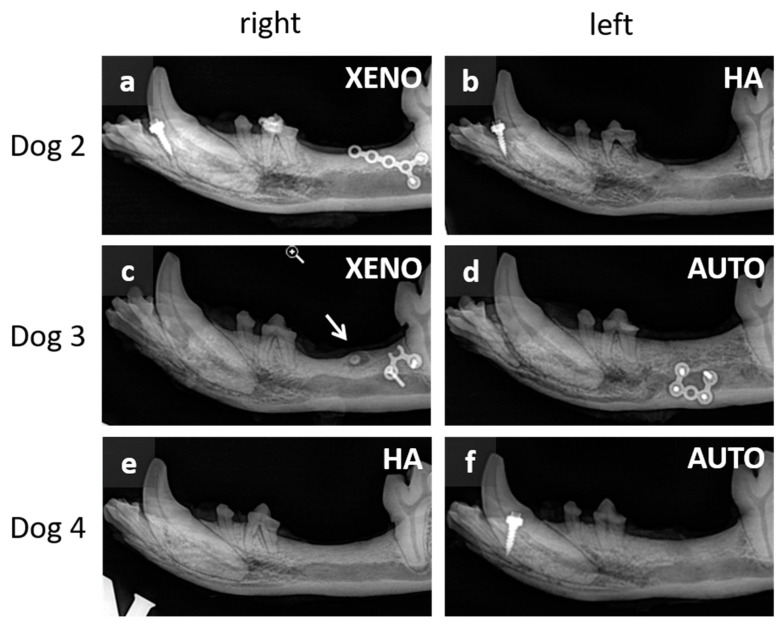
X-ray images of resected mandibles 30 weeks after implantation. (**a**,**c**,**e**): right side of the Dog 2, Dog 3, and Dog 4, respectively; (**b**,**d**,**f**): left side of the Dog 2, Dog 3, and Dog 4. XENO: xenograft; HA: hydroxyapatite; AUTO: autograft. Note that no residuals of BRMs were detectable. The arrow shows an encapsulated root fragment.

Clinically, no residual defects could be observed independent of dogs and BRM. X-rays allowed a more differentiated assessment (Figure 5). Generally, no residual BRM was seen in any jaw of the remaining animals, meaning that bone remodeling had occurred. Dog 2 showed an interruption of the crestal corticalis on the left side of the mandible (augmentation with nanocrystalline HA). In the same animal, more bone above the mandibular canal was seen on the right side (augmented with bovine XENO) when compared with the left side. The alveolar bone levels on the left side (augmentation with AUTO) were comparably higher than on the right side (augmentation with bovine XENO) in Dog 3. Furthermore, an encapsulated root fragment was seen on the right side of the mandible in this dog. A higher alveolar bone level was detected in Dog 4, where augmentation occurred with autogenous bone (AUTO). The alveolar bone level was more pronounced above the mandibular canal when compared with the right side (augmentation with nanocrystalline HA). Radiologically, the bony structures of autogenous bone (AUTO) were similar (Dog 3 and 4 left side). The right jaw in Dog 4 showed a more compact structure. Radiologically, this was similar to the area where bovine XENO was augmented in Dog 3.

### 3.2. Periodontal Gap Measurements

**Figure 6:**
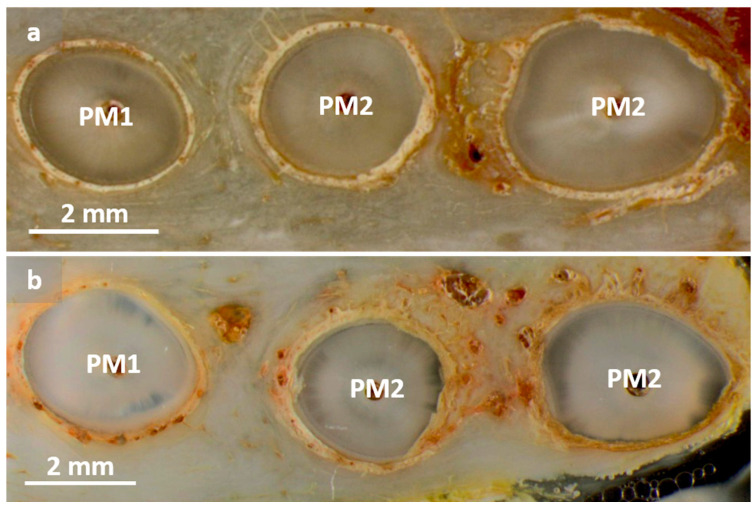
Exemplary light microscopic images of periodontal gaps of the single root of PM1 and the mesial and distal root of PM2: (**a**) Dog 3 left side; (**b**) Dog 2 right side. The periodontal gaps of the single roots of PM1 show smooth and uniform configurations in both animals. Periodontal gaps of mesial and distal roots of PM2 were found to be much wider and irregularly shaped with protuberances around the entire root.

The measurements were taken to check the behavior of a tooth located in the immediate vicinity of the augmented surface and subjected to orthodontic forces. Figure 6 includes two examples of LM images showing the root of PM1, as well as the mesial and distal root of PM2. The edges of both periodontal gaps of PM1 are smooth, and they are uniformly wide around the single tooth. Compared with that for the mesial and distal roots of PM2, much wider periodontal gaps were observed. A series of protuberances were seen around the entire root, making the periodontal gaps of PM2 very irregular.

**Figure 7:**
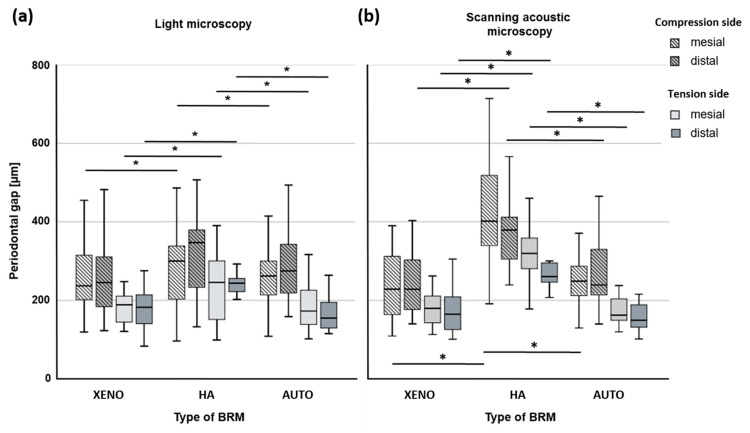
Periodontal gap dimensions (mean and SD) from both roots of PM2, which was orthodontically moved into the augmented critical-sized defect. Periodontal gaps were measured at three points on the compression and tension sides using light microscopic images (**a**), as well as scanning acoustic images (**b**). Mesial and distal roots of PM2 were considered separately. XENO: xenograft; HA: hydroxyapatite; AUTO: autograft; * indicates significant differences between BRMs (*p* ≤ 0.05).

Independent of the type of BRM and root position (mesial, distal), measurements of periodontal gaps using LM images were significantly greater on the compression side when compared with periodontal gaps on the tension side (XENO: 0.25 ± 0.09 µm vs. 0.19 ± 0.05 µm; HA: 0.31 ± 0.13 µm vs. 0.23 ± 0.09 µm; C: 0.27 ± 0.11 µm vs. 0.18 ± 0.06 µm; *p* ≤ 0.05) (Figure 7a). The same was found in SAM images (XENO: 0.24 ± 0.08 µm vs. 0.18 ± 0.06 µm; HA: 0.39 ± 0.14 µm vs. 0.32 ± 0.08 µm; C: 0.27 ± 0.10 µm vs. 0.17 ± 0.04 µm; *p* ≤ 0.05). Considering two different roots in PM2 (mesial, distal), there was no difference between the periodontal gap width on the compression side or tension side except for the mesial root measurement for HA using SAM images (0.35 ± 0.12 µm vs. 0.32 ± 0.06 µm; *p* = 0.22) (Figure 7b). The type of BRM had an influence on the dimension of the periodontal gap. While bovine XENO and autogenous bone (AUTO) lead to similarly dimensioned gap widths, nanocrystalline HA caused different gap widths independent of the microscopic method. Using LM images only, the compression side of the distal root showed no different periodontal gap widths for HA compared with XENO and AUTO. However, the periodontal gaps of the corresponding mesial root, as well as the tension side of both roots, were significantly wider for nanocrystalline HA (*p* ≤ 0.05). SAM images led to a similar result. HA reached the highest values compared with bovine XENO and autogenous bone (AUTO) independent of root position and side of the root (*p* ≤ 0.05). There is an influence of the microscopic method on the results of the periodontal gaps in the case of nanocrystalline HA. For the compression side of the distal root (LM: 0.30 ± 0.12 µm; SAM: 0.40 ± 0.14 µm; *p* ≤ 0.05) and for the tension side of the mesial root (LM: 0.23 ± 0.09 µm; SAM: 0.33 ± 0.07 µm; *p* ≤ 0.05), SAM images revealed significantly greater periodontal gaps when compared with LM images.

### 3.3. Proportion of Bone, Osteoid, and Bone Marrow

**Figure 8:**
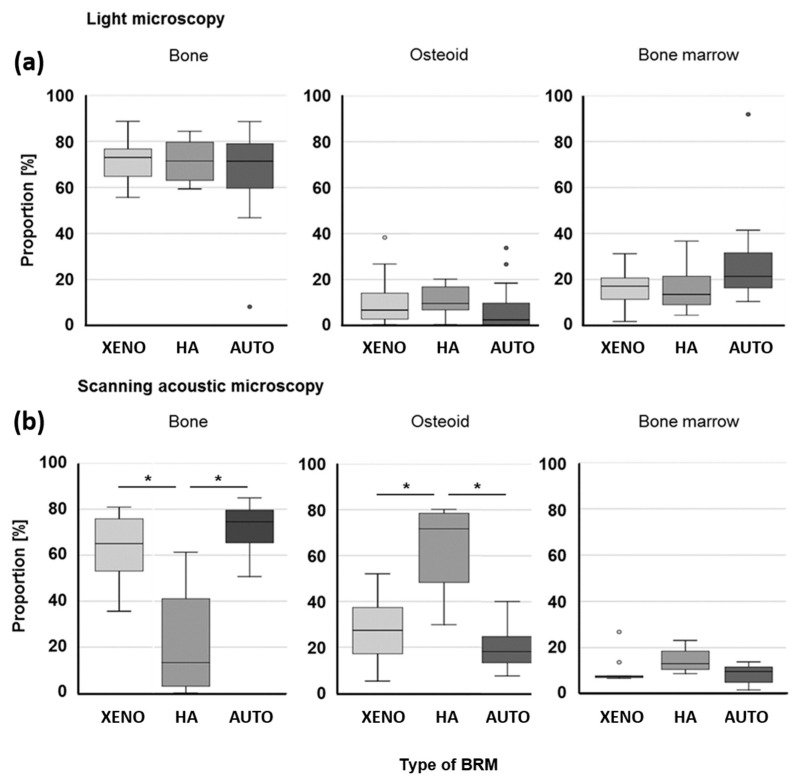
Proportion of bone, osteoid, and bone marrow of the mesial and distal ROI shown as boxplots of light microscopy (**a**) and scanning acoustic microscopy (**b**). XENO: xenograft, HA: hydroxyapatite, AUTO: autograft. * indicates significant differences (*p* ≤ 0.05).

Measurements of new bone formation were performed comparatively using either light microscopy (LM) or scanning acoustic microscopy (SAM) (Figure 8). Both methods of analysis revealed a relatively low proportion of bone marrow independent of the type of BRM (LM: 20.9 ± 13.9%; SAM: 9.8 ± 5.4%; *p* ≤ 0.05). Only the results for autogenous bone (AUTO) differ significantly between LM and SAM (LM: 26.9 ± 22.2%; SAM: 8.5 ± 4.2%; *p* ≤ 0.05). While the LM evaluation also determined a rather small proportion for osteoid (9.1 ± 9.5%), SAM showed significantly higher proportions (29.4 ± 19.4%; *p* ≤ 0.05). LM values for separate BRMs were significantly lower in comparison with the SAM values (XENO: 10.1 ± 11.5% vs. 27.3 ± 13.3%; HA: 6.0 ± 4.3% vs. 63.3 ± 23.1%; AUTO: 4.8 ± 9.5% vs. 20.3 ± 9.5%; *p* ≤ 0.05). The values for the proportion of bone of the bovine xenograft (XENO) and autogenous bone (AUTO) were not significantly different between LM and SAM (XENO: 74.0 ± 9.9% vs. 63.1 ± 15.0%; AUTO: 68.2 ± 22.4% vs. 71.2 ± 11.1%). However, using LM images, values for the nanocrystalline hydroxyapatite (HA) were distinctly higher compared with SAM measurements (75.5 ± 10.3% vs. 22.1 ± 27.5%; *p* ≤ 0.05). The values for HA evaluated with SAM are conspicuous in terms of the quantity of osteoid and mineralized bone when compared with XENO and AUTO. The HA values for bone and osteoid are also striking in comparison with the LM results.

## 4. Discussion

This study on beagle dogs investigated the changes in the width and shape of the periodontal gaps of second premolar roots, which were orthodontically moved into critical-sized defects after augmentation with different BRMs. Autogenous bone graft, as the gold standard, served as the control. Replacement of autogenous bone grafts in critical-sized defects by BRMs would be particularly beneficial in the field of maxillofacial surgery in patients with cleft lip, alveolus, and palate who might need an alveolar bone graft before adjacent teeth can be moved into the former cleft area.

Surgical interventions in dogs went without complications, as did the recovery process of the animals. The exact cause of Dog 1’s fractured mandible during the night remains unknown. As a result of the fracture, physiological feeding of the animal was no longer possible, so it had to be sacrificed immediately. The dimension of a critical-sized defect weakens the bone, making it much more likely to fracture.

A good healing process of critical-sized defects augmented with autogenous bone was expected. Other studies have also shown that it is advantageous to use it in large bony defects, even outside of dental applications [20,21]. Studies showed that the materials tested in this study have already demonstrated their good suitability. The radiographic results of the present study support the findings of other studies [22,23,24,25,26,27] that bovine xenograft and hydroxyapatite are suitable as BRMs. The suitability of these materials before OTM has also been demonstrated in a number of studies [28,29,30,31,32]. In contrast to the present study, in these studies, extraction sockets were created and subsequently filled. A neighboring tooth was then moved into the bony defect. The authors confirmed that teeth could be successfully moved into these augmented areas. The measurements taken in the present study did not allow a direct statement on OTM. Changes in the size and shape of the periodontal gap on the tension and compression side indicate that force application during tooth movement initiates bone resorption and bone formation processes leading to OTM. Histomorphometric analysis (Figure 8) underlined the formation of functional bone. However, bone formation in the nanocrystalline HA resulted in compact bone to such an extent that root resorption of PM2 was observed in both jaws filled with HA. From an orthodontic point of view, this points to the fact that nanocrystalline HA is a rather unsuitable material for augmentation of critical-sized defects prior to teeth being moved into the former defect area. A study by Seifi et al. [31] in dogs found no difference in the proportion of root resorption between HA-filled and non-intervened artificial sockets, but even the non-intervened group showed almost 20% root resorption in the mandible after 2 months. A complete comparability of the studies is not given. It is likely that the granules used by Seifi et al. [31] behave differently and induce different cellular responses than the block used in the present study. In a comparative study by Troedhan et al. [23], the insertion torque values for dental implants in nanocrystalline HA were significantly higher than in bovine xenografts. This suggests that OTM encounters more resistance in HA and, consequently, a higher risk of root resorption compared with bovine xenografts. Gao et al. noted that the conflicting results regarding the correlation between root resorption and bone density indicated the need for further investigation [33].

Our results show a response of the periodontal gap to the applied forces for the graft materials XENO and HA. The measurements revealed lower values for AUTO, XENO, and HA on the tension side, and both LM and SAM showed significantly greater gaps for nanocrystalline HA (Figure 7). The experimental design does not allow us to draw conclusions about the relationship between bone resorption and bone formation. However, the differently dimensioned periodontal gaps on the tension and the compression sides indicated an OTM due to force application. Comparisons with the literature suggest that OTM occurred during 6 months of force application in the present study [28,31,32]. Tanimoto et al. and Abe et al. reported a distance of tooth movement after 6 months of force application of about 6 mm using carbonated HA as well as carbonated HA and deproteinized bovine bone mineral BioOss^®^ (XENO), respectively [28,32].

OTM is a process triggered by the application of force on teeth, resulting in aseptic inflammation [1,2,4]. This results in a highly complex cellular response in which, according to the Biphasic Theory [2], which is based on the pressure–tension theory [4], two processes take place. Alikhani et al. described that “the Catabolic Phase precedes the Anabolic Phase, with distinct cellular and molecular events establishing the limits for each phase”, with both phases occurring on the compression and tension side and at the same time [2]. This will result in a larger periodontal gap on the compression side, while the gap on the tension side will be comparatively smaller. This, in turn, allows the tooth to move in the direction of the applied force. The periodontal gap measurements of the present study confirm the development of the situation described nearly 6 months after the application of the orthodontic force. Based on the situation in the first few days after the start of OTM inducement, the PDL seems to undergo a fundamental restructuring in the following months. Studies that examined both the compression and tension side of the moved teeth within the first 3 to 14 days found a compression of the PDL, a narrowing of the periodontal gap on the compression side of the tooth, and a widening of the periodontal gap on the tension side [10,13,15]. The sustained force application in the present study caused a widening of the PDL on the compression side when compared with the tension side. However, the bone remodeling process appears to be balanced, as described in the Biphasic Theory [2]. Otherwise, it would be assumed that a narrower periodontal gap is still present on the compression side.

## 5. Conclusions

This is the first insight into the long-term effects of changes in size and shape of the periodontal gap in teeth that were moved into critical-sized defects after augmentation with different BRMs. It provides important and complementary information on bone remodeling processes following tooth movement into augmented areas of greater extent. Of course, the small number of animals used in this study allows only a cautious interpretation of the presented results. However, there are now first indications that over the course of months, the compression side is no longer a classical side of compression, as described in the pressure–tension theory [4]. To understand the changes—in terms of time and process—further studies are needed.

## Figures and Tables

**Table 1:** Dimensions of all critical-sized defects in mm (length × height × depth).

Side of the Jaw	Dog 1	Dog 2	Dog 3	Dog 4
Right	29 × 9 × 10	26 × 9 × 8	24 × 9 × 10	26 × 9 × 8
Left	28 × 8 × 10	26 × 10 × 8	26 × 10 × 6	25 × 7 × 8

## Data Availability

The raw data supporting the conclusions of this article will be made available by the authors on request.

## Supplementary Materials

The following supporting information can be downloaded at https://www.mdpi.com/article/10.3390/dj12120386/s1, Text S1: Ethics note.
