# Supplementary material for: Changes in the Periodontal Gap After Long-Term Tooth Movement into Augmented Critical-Sized Defects in the Jaws of Beagle Dogs
Source: Dent J (Basel). 2024 Nov 26;12(12):386. doi: 10.3390/dj12120386 (PMC11674691; doi:10.3390/dj12120386)
Supplement: Supplementary file 1 [file dentistry-12-00386-s001.zip › dentistry-3116474-supplementary.pdf]

Ethic note

Animal care and all experimental procedures were approved by the Institutional Review Board, the State Office for Agriculture, Food Safety and Fisheries of the federal state of Mecklenburg-Vorpommern (Rostock, Germany, Number 7221.3-1.1-064/11, 2011/07/08). The study was conducted in 2012 at the University Medical Centre Rostock, Germany.

Four beagle dogs were used in this prospective animal study. Following the extraction of two teeth, a critical-sized defect was created and filled with bone replacement materials. The distalization of an adjacent tooth began after 7 weeks of healing. The dogs were sacrificed after a further 23 weeks.
